# Assessing fragility of statistically significant findings from randomized controlled trials assessing pharmacological therapies for opioid use disorders: a systematic review

**DOI:** 10.1186/s13063-024-08104-x

**Published:** 2024-04-27

**Authors:** Leen Naji, Brittany Dennis, Myanca Rodrigues, Monica Bawor, Alannah Hillmer, Caroul Chawar, Eve Deck, Andrew Worster, James Paul, Lehana Thabane, Zainab Samaan

**Affiliations:** 1 Department of Family Medicine, David Braley Health Sciences Centre, McMaster University, 100 Main St W, 3rdFloor, Hamilton, ON L8P 1H6 Canada; 2 Department of Health Research Methods, Evidence, and Impact, McMaster University, Hamilton, ON Canada; 3 Department of Medicine, Montefiore Medical Center, New York, NY USA; 4 Department of Medicine, McMaster University, Hamilton, ON Canada; 5 Department of Medicine, University of British Columbia, Vancouver, Canada; 6 Department of Medicine, Imperial College Healthcare NHS Trust, London, UK; 7 Department of Psychiatry and Behavaioral Neurosciences, McMaster University, Hamilton, ON Canada; 8 Physician Assistant Program, University of Toronto, Toronto, ON Canada; 9 Department of Family Medicine, Western University, London, ON Canada; 10 Department of Anesthesia, McMaster University, Hamilton, ON Canada; 11 Biostatistics Unit, Research Institute at St Joseph’s Healthcare, Hamilton, ON Canada; 12 Department of Psychiatry and Behavioral Neurosciences, McMaster University, Hamilton, ON Canada

**Keywords:** Fragility index, Opioid use disorder, Research methods, Randomized controlled trials, Critical appraisal, Systematic review

## Abstract

**Background:**

The fragility index is a statistical measure of the robustness or “stability” of a statistically significant result. It has been adapted to assess the robustness of statistically significant outcomes from randomized controlled trials. By hypothetically switching some non-responders to responders, for instance, this metric measures how many individuals would need to have responded for a statistically significant finding to become non-statistically significant. The purpose of this study is to assess the fragility index of randomized controlled trials evaluating opioid substitution and antagonist therapies for opioid use disorder. This will provide an indication as to the robustness of trials in the field and the confidence that should be placed in the trials’ outcomes, potentially identifying ways to improve clinical research in the field. This is especially important as opioid use disorder has become a global epidemic, and the incidence of opioid related fatalities have climbed 500% in the past two decades.

**Methods:**

Six databases were searched from inception to September 25, 2021, for randomized controlled trials evaluating opioid substitution and antagonist therapies for opioid use disorder, and meeting the necessary requirements for fragility index calculation. Specifically, we included all parallel arm or two-by-two factorial design RCTs that assessed the effectiveness of any opioid substitution and antagonist therapies using a binary primary outcome and reported a statistically significant result. The fragility index of each study was calculated using methods described by Walsh and colleagues. The risk of bias of included studies was assessed using the Revised Cochrane Risk of Bias tool for randomized trials.

**Results:**

Ten studies with a median sample size of 82.5 (interquartile range (IQR) 58, 179, range 52–226) were eligible for inclusion. Overall risk of bias was deemed to be low in seven studies, have some concerns in two studies, and be high in one study. The median fragility index was 7.5 (IQR 4, 12, range 1–26).

**Conclusions:**

Our results suggest that approximately eight participants are needed to overturn the conclusions of the majority of trials in opioid use disorder. Future work should focus on maximizing transparency in reporting of study results, by reporting confidence intervals, fragility indexes, and emphasizing the clinical relevance of findings.

**Trial registration:**

PROSPERO CRD42013006507. Registered on November 25, 2013.

**Supplementary Information:**

The online version contains supplementary material available at 10.1186/s13063-024-08104-x.

## Introduction

Opioid use disorder (OUD) has become a global epidemic, and the incidence of opioid related fatality is unparalleled to the rates observed in North America, having climbed 500% in the past two decades [1, 2]. There is a dire need to identify the most effective treatment modality to maintain patient engagement in treatment, mitigate high risk consumption patterns, as well as eliminate overdose risk. Numerous studies have aimed to identify the most effective treatment modality for OUD [3–5]. Unfortunately, this multifaceted disease is complicated by the interplay between both neurobiological and social factors, impacting our current body of evidence and clinical decision making. Optimal treatment selection is further challenged by the rising number of pharmacological opioid substitution and antagonist therapies (OSAT) [6]. Despite this growing body of evidence and available therapies, we have yet to arrive to a consensus regarding the best treatment modality given the substantial variability in research findings and directly conflicting results [6–9]. More concerning, international clinical practice guidelines rely on out-of-date systematic review evidence to inform guideline development [10]. In fact, these guidelines make strong recommendations based on a fraction of the available evidence, employing trials with restrictive eligibility criteria which fail to reflect the common OUD patients seen in clinical practice [10].

A major factor hindering our ability to advance the field of addiction medicine is our failure to apply the necessary critical lens to the growing body of evidence used to inform clinical practice. While distinct concerns exist regarding the external validity of randomized trials in addiction medicine, the robustness of the universally recognized “well designed” trials remains unknown [10]. The reliability of the results of clinical trials rests on not only the sample size of the study but also the number of outcome events. In fact, a shift in the results of only a few events could in theory render the findings of the trial null, impacting the traditional hypothesis tests above the standard threshold accepted as “statistical significance.” A metric of this fragility was first introduced in 1990, known formally as the fragility index (FI) [11]. In 2014, it was adapted for use as a tool to assess the robustness of findings from randomized controlled trials (RCTs) [12]. Briefly, the FI determines the minimum number of participants whose outcome would have to change from non-event to event in order for a statistically significant result to become non-significant. Larger FIs indicate more robust findings [11, 13]. Additionally, when the number of study participants lost to follow-up exceeds the FI of the trial, this implies that the outcome of these participants could have significantly altered the statistical significance and final conclusions of the study. The FI has been applied across multiple fields, often yielding similar results such that the change in a small number of outcome events has been powerful enough to overturn the statistical conclusions of many “well-designed” trials [13].

The concerning state of the OUD literature has left us with guidelines which neither acknowledge the lack of external validity and actually go so far as to rank the quality of the evidence as good, despite the concerning limitations we have raised [10]. Such alarming practices necessitate vigilance on behalf of methodologists and practitioners to be critical and open to a thorough review of the evidence in the field of addiction medicine [12]. Given the complex nature of OUD treatment and the increasing number of available therapies, concentrated efforts are needed to ensure the reliability and internal validity of the results of clinical trials used to inform guidelines. Application of the FI can serve to provide additional insight into the robustness of the evidence in addiction medicine. The purpose of this study is to assess the fragility of findings of RCTs assessing OSAT for OUD.

## Methods

### Systematic review protocol

We conducted a systematic review of the evidence surrounding OSATs for OUD [5]. The study protocol was registered with PROSPERO a priori (PROSPERO CRD42013006507). We searched Medline, EMBASE, PubMed, PsycINFO, Web of Science, and Cochrane Library for relevant studies from inception to September 25, 2021. We included all RCTs evaluating the effectiveness of any OSAT for OUD, which met the criteria required for FI calculation. Specifically, we included all parallel arm or two-by-two factorial design RCTs that allocated patients at a 1:1 ratio, assessed the effectiveness of any OSAT using a binary primary or co-primary outcome, and reported this outcome to be statistically significant (*p* < 0.05).

All titles, abstracts, and full texts were screened for eligibility by two reviewers independently and in duplicate. Any discrepancies between the two reviewers were discussed for consensus, and a third reviewer was called upon when needed.

### Data extraction and risk of bias assessment (ROB)

Two reviewers extracted the following data from the included studies in duplicate and independently using a pilot-tested excel data extraction sheet: sample size, whether a sample size calculation was conducted, statistical test used, primary outcome, number of responders and non-responders in each arm, number lost to follow-up, and the *p*-value. The 2021 Thomson Reuters Journal Impact Factor for each included study was also recorded. The ROB of included studies for the dichotomous outcome used in the FI calculation was assessed using the Revised Cochrane ROB tool for randomized trials [14]. Two reviewers independently assessed the included studies based on the following domains for potential ROB: randomization process, deviations from the intended interventions, missing outcome data, measurement of the outcome, and selection of the reported results.

### Statistical analyses

Study characteristics were summarized using descriptive statistics. Means and standard deviations (SD), as well as medians and interquartile ranges (IQR: Q_25_, Q_75_) were used as measures of central tendency for continuous outcomes with normal and skewed distributions, respectively. Frequencies and percentages were used to summarize categorical variables. The FI was calculated using a publicly available free online calculator, using the methods described by Walsh et al. [12, 15] In summary, the number of events and non-events in each treatment arm were entered into a two-by-two contingency table for each trial. An event was added to the treatment arm with the smaller number of events, while subtracting a non-event from the same arm, thus keeping the overall sample size the same. Each time this was done, the two-sided *p*-value for Fisher’s exact test was recalculated. The FI was defined as the number of non-events that needed to be switched to events for the *p*-value to reach non-statistical significance (i.e., ≥0.05).

We intended to conduct a linear regression and Spearman’s rank correlations to assess the association between FI and journal impact factor, study sample size, and number events. However, we were not powered to do so given the limited number of eligible studies included in this review and thus refrained from conducting any inferential statistics.

## Results

We followed the Preferred Reporting Items for Systematic Reviews and Meta-Analyses (PRISMA) guidelines for reporting (see Supplementary Material) [16].

### Study selection

**Fig. 1:**
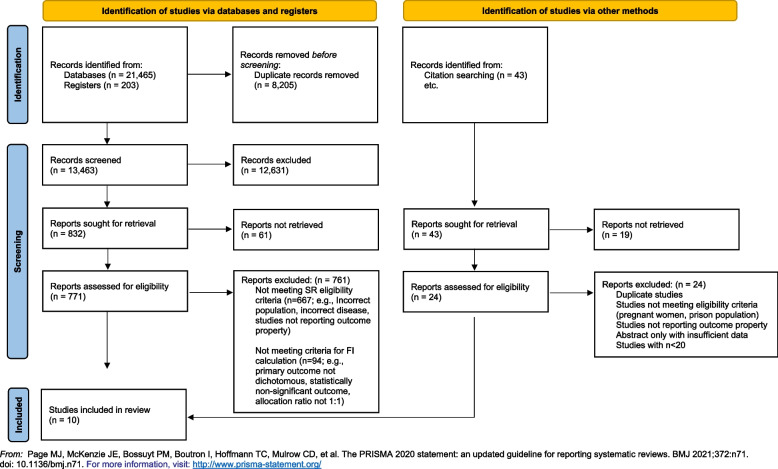
PRISMA flow diagram delineating study selection

Our search yielded 13,463 unique studies, of which 104 were RCTs evaluating OSAT for OUD. Among these, ten studies met the criteria required for FI calculation and were included in our analyses. Please refer to Fig. 1 for the search results, study inclusion flow diagram, and Table 1 for details on included studies.

**Table 1:** Characteristics of included RCTs, in order of increasing FI (*k* = 10)

Authors	Year	Outcome	Total sample size [*n* (*n*_arm 1_, *n*_arm 2_)]	No. lost to follow-up (%)	*p*-value	Journal impact factor	Fragility index	Overall ROB
Krupitsky et al. [22]	2004	Treatment retention	52 (25, 27)	0 (0)^a^	<0.05	3.77	1	Low
Fischer et al. [25]	1999	Treatment retention	60 (31, 29)	0 (0)^a^	0.03	6.53	2	Some concerns
Petitijean et al. [17]	2001	Treatment retention	58 (31, 27)	0 (0)^a^	0.002	4.50	4	Low
Krook et al. [23]	2002	Treatment retention	106 (51, 55)	7 (6.6)^a^	<0.001	6.53	6	Low
Sees et al. [18]	2000	Treatment retention	179 (91, 88)	0 (0)^a^	0.01	56.27	7	Some concerns
Kakko et al. [19]	2003	Treatment retention	40 (20, 20)	0 (0)^a^	0.0001	79.32	8	Low
Yancovitz et al. [26]	1991	Heroin use	301 (94, 75)	132 (43.9)	<0.001	9.30	11	High
Hartnoll et al. [24]	1980	Treatment retention	96 (52, 44)	8 (8.3)^a^	0.001	8.48	12	Low
Hulse et al. [21]	2009	Maintaining therapeutic level of naltrexone in blood	69 (34, 35)	9 (13.0)	<0.001	8.48	15	Low
Oviedo-Joekes et al. [20]	2009	Treatment retention	226 (111, 115)	6 (2.7)^a^	<0.001	91.25	26	Low

^a^Should be interpreted with caution as primary dichotomous outcome is treatment retention, thus we only report on losses to follow-up for known reasons unrelated to treatment, as it is otherwise difficult to distinguish loss to follow-up from the outcome treatment retention

### Characteristics of included studies

The included studies were published between 1980 and 2018, in eight different journals with a median impact factor of 8.48 (IQR 6.53–56.27, range 3.77–91.25). Four studies reported on a calculated sample size [17–20], and only one study specified that reporting guidelines were used [21]. Treatment retention was the most commonly reported primary outcome (*k* = 8). The median sample size of included studies was 82.5 (IQR 58–179, range 52–226).

Overall ROB was deemed to be low in seven studies [17, 19–24], have some concerns in two studies [18, 25], and be high in one study [26] due to a high proportion of missing outcome data that was not accounted for in the analyses. We present a breakdown of the ROB assessment of the included studies for the dichotomous outcome of interest in Table 2.

**Table 2:** Risk of bias of included RCTs for dichotomous outcome of interest (*n* = 10)

Dimension assessed	Low No. (%)	Some concerns No. (%)	High No. (%)
ROB arising from randomization process	8 (80)	2 (20)	0
ROB due to deviations from the intended intervention	8 (80)	2 (20)	0
ROB due to missing outcome data	9 (90)	0	1 (10)
ROB in measurement of the outcome	10 (100)	0	0
ROB in selection of the reported result	10 (100)	0	0
Overall ROB	7 (70)	2 (20)	1 (10)

### Fragility index

The median FI of included studies was 7.5 (IQR 4–12; range 1–26). The FI of individual studies is reported in Table 1. The number of participants lost to follow-up exceeded the FI in two studies [23, 26]. We find that there is a relatively positive correlation between the FI and sample size. However, no clear correlation was appreciated between FI and journal impact factor or number of events.

## Discussion

This is the first study to evaluate the FI in the field of addiction medicine, and more specifically in OUD trials. Among the ten RCTs evaluating the OSAT for OUD, we found that, in some cases, changing the outcome of one or two participants could completely alter the study’s conclusions and render the results statistically non-significant.

We compare our findings to those of Holek et al.*,*wherein they examined the mean FI across all reviews published in PubMed between 2014 and 2019 that assessed the distribution of FI indices, irrespective of discipline (though none were in addiction medicine) [13]. Among 24 included reviews with a median sample size of 134 (IQR 82, 207), they found a mean FI of 4 (95% CI 3, 5) [13]. This is slightly lower than our calculated our median FI of 7.5 (IQR 4–12; range 1–26). It is important to note that half of the reviews included in the study by Holek et al. were conducted in surgical disciplines, which are generally subjected to more limitations to internal and external validity, as it is often not possible to conceal allocation, blind participants, or operators, and the intervention is operator dependent. [27] To date, no study has directly applied FI to the findings of trials in OUD. In the HIV/AIDS literature, however, a population which is commonly shared with addiction medicine due to the prevalence of the comorbidities coexisting, the median fragility across all trials assessing anti-retroviral therapies (*n*= 39) was 6 (IQR = 1, 11) [28], which is more closely related to our calculated FI. Among the included studies, only 3 were deemed to be at high risk of bias, whereas 13 and 20 studies were deemed to be at low and some risk of bias, respectively.

Loss-to-follow-up plays an important role in the interpretation of the FI. For instance, when the number of study participants lost to follow-up exceeds the FI of the trial, this implies that the outcome of these participants could have significantly altered the statistical significance and final conclusions of the study. While only two of the included studies had an FI that was greater than the total number of participants lost to follow-up [23, 26], this metric is less important in our case given the primary outcome assessed by the majority of trials was retention in treatment, rendering loss to follow-up an outcome itself. In our report, we considered participants to be lost to follow-up if they left the study for reasons that were known and not necessarily indicative of treatment failure, such as due to factors beyond the participants, control including incarceration or being transferred to another treatment location.

Findings from our analysis of the literature as well as the application of FI to the existing clinical trials in the field of addiction medicine demonstrates significant concerns regarding the robustness of the evidence. This, in conjunction with the large differences between the clinical population and trial participants of opioid-dependent patients inherent in addiction medicine trials, raises larger concerns as to a growing body of evidence with deficiencies in both internal and external validity. The findings from this study raise important clinical concerns regarding the applicability of the current evidence to treating patients in the context of the opioid epidemic. Are we recommending the appropriate treatments for patients with OUD based on robust and applicable evidence? Are we completing our due diligence and ensuring clinicians and researchers alike understand the critical issues rampant in the literature, including the fragility of the data and misconceptions of *p*-values? Are we possibly putting our patients at risk employing such treatment based on fragile data? These questions cannot be answered until the appropriate re-evaluation of the evidence takes place employing both the use pragmatic trial designs as well as transparent metrics to reflect the reliability and robustness of the findings.

### Strengths and limitations

Our study is strengthened by a comprehensive search strategy, rigorous and systematic screening of studies, and the use of an objective measure to gauge the robustness of studies (i.e., FI). The limitations of this study are inherent in the limitations of the FI. Precisely, that it can only be calculated for RCTs with a 1:1 allocation ratio, a parallel arm or two-by-two factorial design, and a dichotomous primary outcome. As a result, 94 RCTs evaluating OSAT for OUD were excluded for not meeting these criteria (Fig. 1). Nonetheless, the FI provides a general sense of the robustness of the available studies, and our data reflect studies published across almost four decades in journals of varying impact factor.

### Future direction

This study serves as further evidence for the need of a shift away from *p*-values [29, 30]. Although there is increasingly a shift among statisticians to shift away from relying on statistical significance due to its inability to convey clinical importance [31], this remains the simplest way and most commonly reported metric in manuscripts. *p*-values provide a simple statistical measure to confirm or refute a null hypothesis, by providing a measure of how likely the observed result would be if the null hypothesis were true. An arbitrary cutoff of 5% is traditionally used as a threshold for rejecting the null hypothesis. However, a major drawback of the *p*-value is that it does not take into account the effect size of the outcome measure, such that a small incremental change that may not be clinically significant may still be statistically significant in a large enough trial. Contrastingly, a very large effect size that has biological plausibility, for instance, may not reach statistical significance if the trial size is not large enough [29, 30]. This is highly problematic given the common misconceptions surrounding the *p*-value. Increasing emphasis is being placed on the importance of transparency in outcome reporting, and the reporting of confidence intervals to allow the reader to gauge the uncertainty in the evidence, and make a clinically informed decision about whether a finding is clinically significant or not. It has also been recommended that studies report FI where possible to provide readers with a comprehensible way of gauging the robustness of their findings [12, 13]. There is a strive to make all data publicly available, allowing for replication of study findings as well as pooling of data among databases for generating more robust analyses using larger pragmatic samples [32]. Together, these efforts aim to increase transparency of research and facilitate data sharing to allow for stronger and more robust evidence to be produced, allowing for advancements in evidence-based medicine and improvements in the quality of care delivered to patients.

## Conclusion

Our results suggest that approximately eight participants are needed to overturn the conclusions of the majority of trials in addiction medicine. Findings from our analysis of the literature and application of FI to the existing clinical trials in the field of addiction medicine demonstrates significant concerns regarding the overall quality and specifically robustness and stability of the evidence and the conclusions of the trials. Findings from this work raises larger concerns as to a growing body of evidence with deficiencies in both internal and external validity. In order to advance the field of addiction medicine, we must re-evaluate the quality of the evidence and consider employing pragmatic trial designs as well as transparent metrics to reflect the reliability and robustness of the findings. Placing emphasis on clinical relevance and reporting the FI along with confidence intervals may provide researchers, clinicians, and guideline developers with a transparent method to assess the outcomes from clinical trials, ensuring vigilance in decisions regarding management and treatment of patients with substance use disorders.

### Supplementary Information

**Supplementary Material 1.**

## Data Availability

All data generated or analyzed during this study are included in this published article (and its supplementary information files).

## Abbreviations

IQR: Interquartile range
OUD: Opioid use disorder
OSAT: Opioid substitution and antagonist therapies
FI: Fragility index
RCTs: Randomized controlled trials
ROB: Risk of bias
SD: Standard deviation
PRISMA: Preferred Reporting Items for Systematic Reviews and Meta-Analyses
